# Comparison of 16-Channel Asymmetric Sleeve Antenna and Dipole Antenna Transceiver Arrays at 10.5 Tesla MRI

**DOI:** 10.1109/TMI.2020.3047354

**Published:** 2021-04-01

**Authors:** Myung Kyun Woo, Lance DelaBarre, Matt Waks, Jingu Lee, Russell Luke Lagore, Steve Jungst, Andrea Grant, Yigitcan Eryaman, Kamil Ugurbil, Gregor Adriany

**Affiliations:** Center for Magnetic Resonance Research (CMRR), University of Minnesota, Minneapolis, MN 55455 USA; Center for Magnetic Resonance Research (CMRR), University of Minnesota, Minneapolis, MN 55455 USA; Center for Magnetic Resonance Research (CMRR), University of Minnesota, Minneapolis, MN 55455 USA; Department of Electrical and Computer Engineering, Seoul National University, Seoul 08826, South Korea, and also with AIRS Medical Inc., Seoul 08788, South Korea; Center for Magnetic Resonance Research (CMRR), University of Minnesota, Minneapolis, MN 55455 USA; Center for Magnetic Resonance Research (CMRR), University of Minnesota, Minneapolis, MN 55455 USA; Center for Magnetic Resonance Research (CMRR), University of Minnesota, Minneapolis, MN 55455 USA; Center for Magnetic Resonance Research (CMRR), University of Minnesota, Minneapolis, MN 55455 USA; Center for Magnetic Resonance Research (CMRR), University of Minnesota, Minneapolis, MN 55455 USA; Center for Magnetic Resonance Research (CMRR), University of Minnesota, Minneapolis, MN 55455 USA

**Keywords:** Cable trap, dipole antenna, deep brain imaging, asymmetric sleeve antenna, ultra-high field magnetic resonance imaging

## Abstract

Multi-element transmit arrays with low peak 10 g specific absorption rate (SAR) and high SAR efficiency (defined as $\left({\text{B}}_{1}^{+}/{\sqrt{\;}}_{\text{peak}}\text{SA}{\text{R}}_{10\text{g}}\right)$ are essential for ultra-high field (UHF) magnetic resonance imaging (MRI) applications. Recently, the adaptation of dipole antennas used as MRI coil elements in multi-channel arrays has provided the community with a technological solution capable of producing uniform images and low SAR efficiency at these high field strengths. However, human head-sized arrays consisting of dipole elements have a practical limitation to the number of channels that can be used due to radiofrequency (RF) coupling between the antenna elements, as well as, the coaxial cables necessary to connect them. Here we suggest an asymmetric sleeve antenna as an alternative to the dipole antenna. When used in an array as MRI coil elements, the asymmetric sleeve antenna can generate reduced peak 10 g SAR and improved SAR efficiency. To demonstrate the advantages of an array consisting of our suggested design, we compared various performance metrics produced by 16-channel arrays of asymmetric sleeve antennas and dipole antennas with the same dimensions. Comparison data were produced on a phantom in electromagnetic (EM) simulations and verified with experiments at 10.5 Tesla (T). The results produced by the 16-channel asymmetric sleeve antenna array demonstrated 28 % lower peak 10 g SAR and 18.6 % higher SAR efficiency when compared to the 16-channel dipole antenna array.

## I. Introduction

**M**AGNETIC resonance imaging (MRI) at ultra-high fields (UHF, defined as ≥7 tesla (T)) are increasingly pursued for biomedical research due to gains in signal-to-noise [1]–[4] and, in some cases, contrast-to-noise ratios (SNR and CNR, respectively) (e.g. [5], [6]). This has led to initiatives pushing human MRI systems to extremely high magnetic field strengths, such as 10.5 T [7], [8]. However, in the UHF regime, the ratio of the wavelength of the radiofrequency (RF) electromagnetic (EM) waves employed for excitation of signals from the water protons in tissue compared to the object size becomes less than one [2], [9]. Consequently, UHF RF coil designs frequently migrate towards far field antenna concepts rather than the near field domain, like those used at current clinical MRI field strengths.

As MRI pushes into the UHF regime, electric (E) and magnetic (B) field amplitude and phase non-uniformities increase over the sample volume, and this leads to non-uniform power deposition and transmit efficiency [4], [9]–[12]. Radiative type antennas [8], [13]–[15], particularly half wavelength (*λ*/2) dipole antennas, have been suggested as building blocks for such UHF transmit arrays and have recently shown promising performance initially for applications in the human torso [7], [14], [16], [17] and recently in the human head [8], [18], [19] enabling improved transmit B_1_ efficiency and minimized power deposition in the imaging target (i.e. specific absorption rate (SAR)). Compared to other RF coil types, such as loops (e.g. [20], [21]) or microstrip type structures [22], [23], dipole antennas show more favorable Poynting vectors and improved B_1_ shimming performance. Dipole antennas also achieve greater penetration depth, however they encounter greater challenges in minimizing the mutual coupling between neighboring elements. For applications in the human torso, this challenge is mitigated by positioning the antenna in consistent close proximity to the body, thus promoting maximal coupling between the sample and the antenna while maintaining consistent coupling between neighboring elements. Similarly, subject-specific stripline coil arrangements in combination with geometric capacitive decoupling schemes had been successfully utilized to support more reliable coil loading for head arrays at 7 T [24]. More recently, in order to minimize coupling for radiative antenna arrays, a number of innovative decoupling techniques have been suggested [25]–[28]. However mutual coupling and radiation remain a significantly problem for human head applications at 10.5 T due to the relatively large and non-uniform gap between the head and the antenna array [27], [29], [30]. The lack of strong coupling to the imaging object encountered for dipole antenna array implementations for human head imaging also results in stronger interaction between the dipole antenna and the coaxial feed cable, which is typically routed in parallel to one leg of the dipole antenna in MR applications. Combined with the interaction among the many coaxial cables in a multi-element array, degraded antenna performance and significant E- and B-field perturbations have been observed [31]–[33].

Here we adapt the sleeve antenna concept for MR imaging at UHF. The “sleeve” of the sleeve antenna concept has the same structure as a cable trap and we can elegantly use this for reducing sheath currents on the coaxial feed cable. We incorporate these floating sleeves [34], [35] into the antenna feed structure and extend the concept towards development of an asymmetric sleeve antenna array for MRI applications in the human head. In this paper, we describe a 16-channel asymmetric sleeve antenna array design for 447 MHz (10.5 T human head ^1^H imaging), which, at the time of this publication, is the highest magnetic field available for human imaging. We compare this asymmetric sleeve antenna array to a dipole antenna array and present validation with EM simulations and 10.5 T MR experiments, demonstrating advantages in ${\text{B}}_{1}^{+}$ efficiency (defined as ${\text{B}}_{1}^{+}$ amplitude in the center of the coil per unit square root power), 10 g SAR, and SAR efficiency (defined as $\left({\text{B}}_{1}^{+}\text{center}/{\sqrt{\;}}_{\text{peak}}\text{SA}{\text{R}}_{10\text{g}}\right)$).

## II. Methods

### A. Antenna Concept and Design of the Asymmetric Sleeve Antenna

**Fig. 1.**
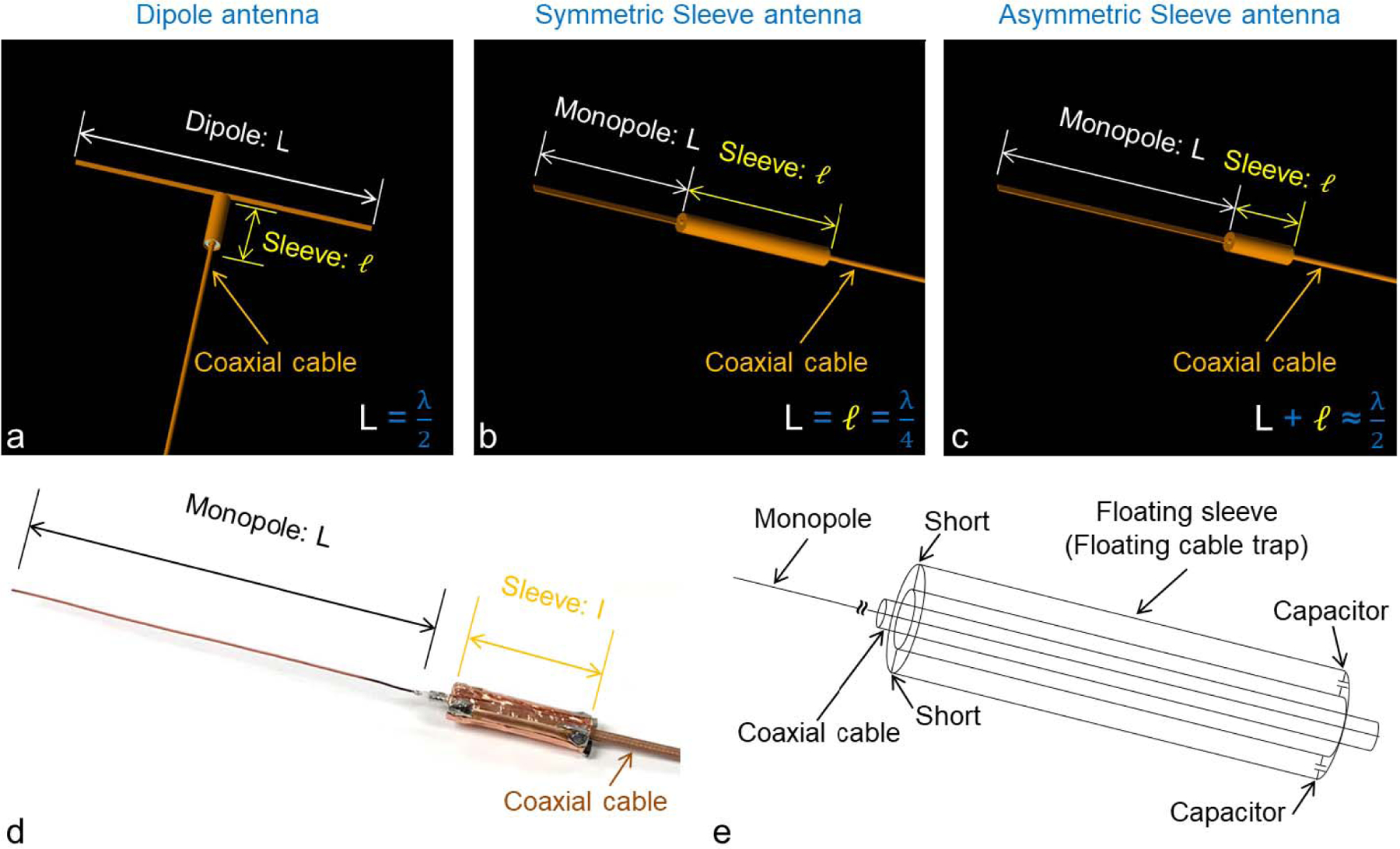
Modification steps involved in converting a dipole antenna (a) to a symmetric sleeve antenna (b) then to an asymmetric sleeve antenna (c). The basic structures of a dipole and a symmetric sleeve antenna are equivalent (b). However, the sleeve antenna is an end-fed structure (b and c) while the dipole antenna is a center-fed structure (a). The sleeve portion of the sleeve antenna is a part of the antenna which acts as the ground. The architecture of the sleeve antenna leads the freedom to modify the length of the antenna part, which consists of the monopole, and sleeve portion, leading to an asymmetric sleeve antenna (c-e). Photograph (d) and schematic diagram (e) of an asymmetric sleeve antenna. The sleeve portion of the antenna is mechanically fixed in position, remains electrically floating without any direct contact to conductors of the coaxial cable.

The 3D modeling of a single element dipole antenna (Fig. 1a), a symmetric sleeve antenna (Fig. 1b) and an asymmetric sleeve antenna (Fig. 1c) are shown above, indicating the evolution from a classical half wave dipole to an asymmetric sleeve antenna. The practical implementation is shown in Fig. 1d; and drawn schematically in Fig. 1e. The basic structure of a sleeve antenna [34]–[38] is configured as a seamless combination of a monopole (= *λ*/4) built from the center conductor of a coaxial cable with one floating cable trap (= *λ*/4) placed over the shield. The resulting structure is equivalent to a dipole antenna since the total structural length of the monopole and associated floating cable trap approximate a half wavelength. The length of the floating cable trap and monopole can be varied as long as the sum of the aforementioned parts remains the same (Fig. 1c–1e). The possibility to vary the length of the monopole and sleeve portions of the antenna adds an essential degree of freedom in the design and supports asymmetrical construction with benefits for the overall antenna layout [37], [38].

Theoretically the current distribution of the half wave dipole and the asymmetric sleeve antenna can be described as

(1) $${\text{I}}_{\text{Dipole}}={\text{I}}_{0}\text{sin}\left(\frac{\beta L}{2}\right)$$

(2) $${\text{I}}_{\text{Sleeve}}={\text{I}}_{0}\text{sin}\left(\beta \left(\frac{L}{2}-h\right)\right)\;\;\;\;={\text{I}}_{0}\text{sin}\left(\beta \ell \right),$$

where I_Dipole_ and I_Sleeve_ are the current of the dipole and asymmetric sleeve antenna at the matching point, respectively. ß is the phase constant associated with the transmission line, L is the length of dipole antenna (Fig. 1a) and *ℓ* is the length of the floating sleeve (Fig. 1c) [38], [39].

In air, the resonant length of a dipole antenna is the sum of the equal length poles. At 447 MHz, this half wavelength is ∼330 mm. For the sleeve antenna, the length of the antenna is the sum of the length of monopole and floating cable trap, which is also approximately one half of the wavelength [36], [37]. Due to dielectric media in close proximity (e.g., human head), the actual effective resonance length of the antenna is shortened to ∼250 mm for both dipole antenna and sleeve antenna.

### B. Construction of the 16-Channel Arrays

**Fig. 2.**
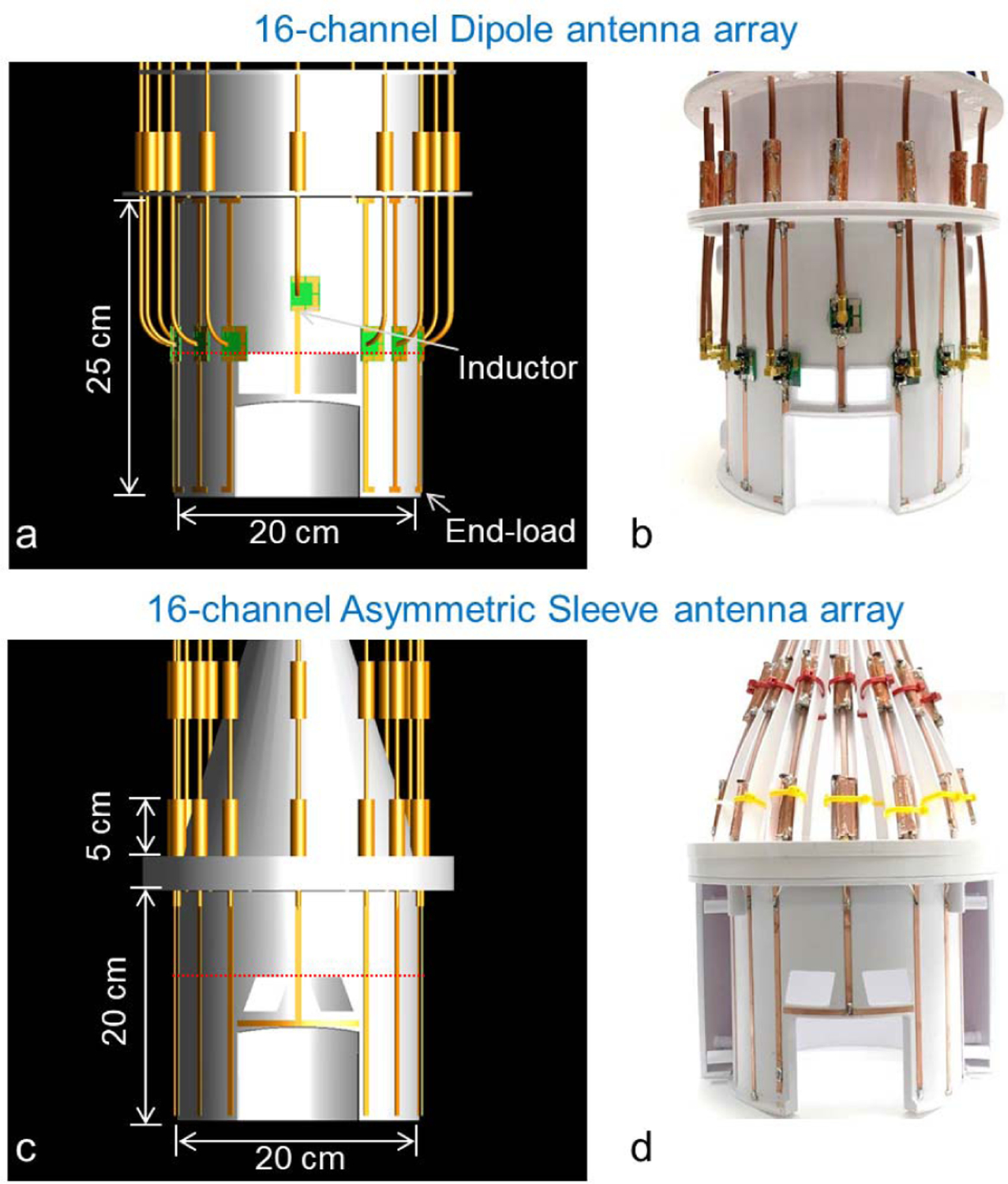
3D modeling (a and c) and photographs (b and d) of the 16-channel dipole and the 16-channel asymmetric sleeve antenna arrays. Importantly, all coaxial cables were included in the simulation to calculate E- and B-fields. Red dotted lines in Fig. 2a and 2c indicate the location of the individual transmit field maps displayed in Fig. 4c and 4f, respectively.

The coil arrays and related coil formers were 3D modeled and the formers were fabricated in-house using a 3D printer (F410, Fusion3 Design, Greensboro, NC, USA). Both the 16-channel dipole (Fig. 2a and 2b) and asymmetric sleeve antenna arrays (Fig. 2c and 2d) were designed and fabricated to the same physical inner dimensions. The array elements were arranged on an elliptically shaped former with a minor axis of 100 mm and major axis of 110 mm. The length of the formers is 250 mm for the dipole antenna array and 200 mm for the asymmetric sleeve antenna array. This geometry results in an arrangement of sixteen antennas with 39 ± 14 mm spacing between individual elements. These arrays use the same elements for B_1_ transmit and receive (i.e. transceive array); the concepts, however, can be extended to transmit only and receive only designs. A 16-channel dipole antenna (length: 250 mm) array with end points were built [15], [40]. This array is mounted on an elliptical shaped holder and the distance from the sample to each antenna varies slightly between elements. This results in variations of the capacitive loading between the sample and individual antenna elements. This variation in sample distance leads to slightly different inductances per elements (from 5 to 17 nH). Fine tuning of individual elements was achieved by adjusting the end points of each dipole antenna.

In the frontal location, tuning inductors were inserted into both legs of the dipole in order to achieve the required physical length reduction while preserving electrical length and subsequent resonance frequency. The use of lattice balun match circuit and a floating cable trap significantly reduced sheath currents for each element of the 16-channel dipole antenna array (Fig. 2a and 2b). The length of each monopole conductor of the 16-channel asymmetric sleeve antenna array was set to 200 mm and combined with the 50 mm floating cable trap as shown in Fig. 2c and 2d.

All floating cable traps were built utilizing 50 mm long 3D printed polyethylene terephthalate glycol-modified (PETG) pipe structures. Each pipe has a 12 mm outer diameter and 5 mm inner diameter which accommodates the RG-400 coaxial feed cable. Two ceramic capacitors (100B series, American Technical Ceramics, Huntington Station, NY, USA) and one variable capacitor (JZ200HV, Knowles Voltronics, Cazenovia, NY, USA) were used to adjust the resonance frequency of the cable traps. Two sets of cable traps were utilized for each sleeve element, with the first set located at the nearest point to the antenna feed point; the second set located another quarter wavelength down the feed cable [34], [35]. The 16-channel dipole antenna array was equipped with the same type of floating cable traps at a quarter wavelength distance from the feed point. These cable traps reduced the coupling between the coaxial cable and dipole antenna. Optimally, cable traps should be located in immediate proximity to the feed point. In practice, however, cable traps are resonant structures that can interact with one of the dipole antenna poles. Thus, in practice it is beneficial to locate the cable traps up the feed coax *λ*/4 from the feed point for the dipole antenna.

### C. Experimental Setup and Bench Measurements

**Fig. 3.**
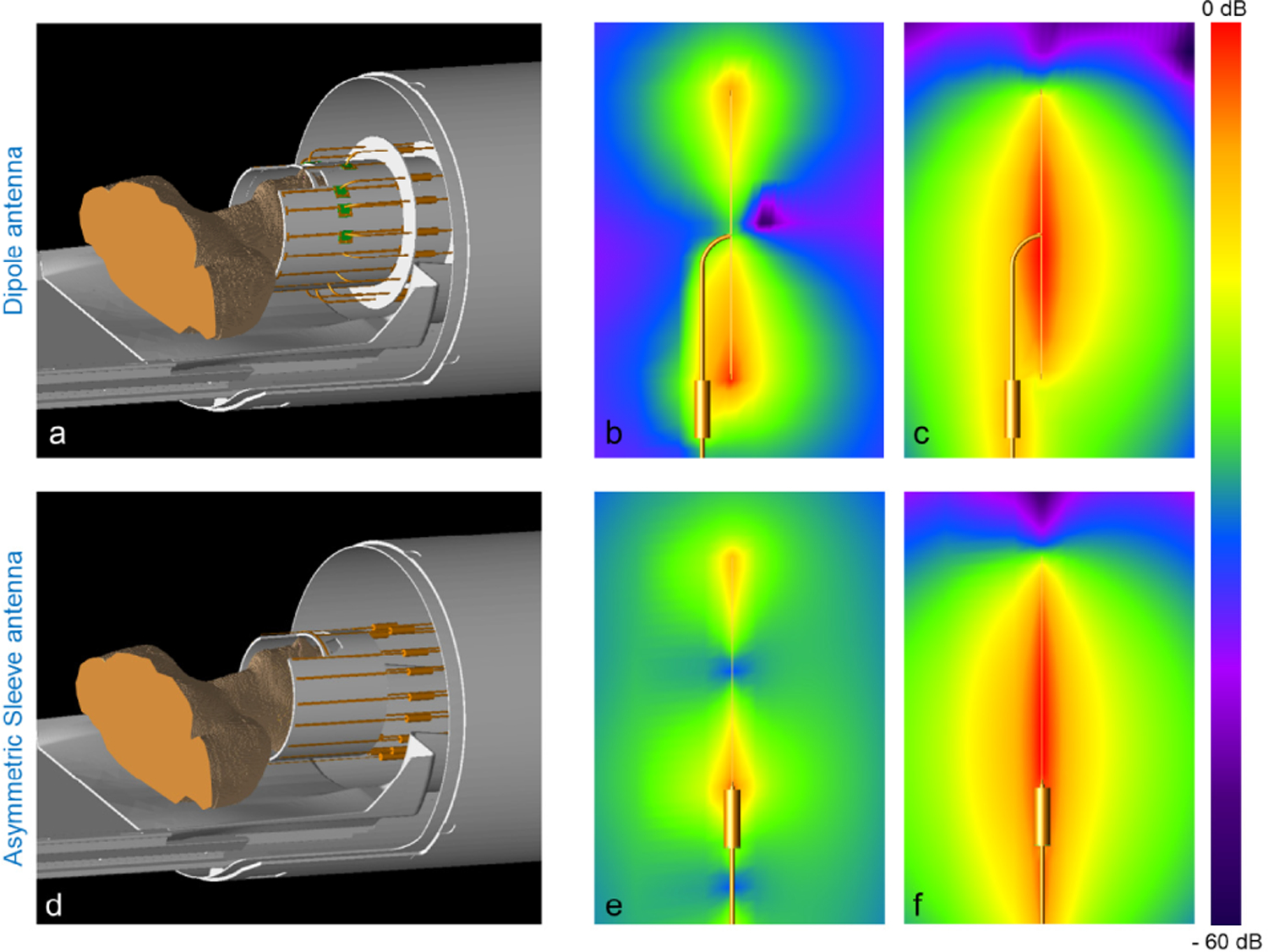
Simulation model of a dipole antenna array (a) and an asymmetric sleeve antenna array (d) with coaxial cables in the bore of the MRI system. For realistic head imaging, coaxial feed cables have to be positioned in parallel alignment with antennas for an in bore setup. Individual E-fields in free space including simulation of the coaxial cable of one dipole antenna (b) and one asymmetric sleeve antenna (e) are shown. Also shown are corresponding B-fields of one dipole antenna (c) and one asymmetric sleeve antenna (f). Note the higher interaction of the dipole antenna array with the center-fed coaxial cable, which results in high E- and B-fields between one pole of a dipole antenna and a coaxial cable (b and c). However, this is notably minimized in the asymmetric sleeve antenna (e and f).

All MR experiments were performed using a 10.5 T / 880 mm whole body magnet (Agilent, Santa Clara, CA, USA) interfaced with a MAGNETOM 10.5 T console (Siemens Healthineers, Erlangen, Germany) equipped with 16 independent parallel transmit (pTx) channels. The pTx system allowed for full control over transmitter phase, amplitude, timing, and waveform. For better comparison with standard systems all data presented here were acquired with equal RF transmit power per channel. A 16-channel transmit/receive interface box (Virtumed, Minneapolis, MN, USA), mounted on the patient table was equipped with in-house built transmit/receive switch modules to connect all array elements to the MRI system as shown in Fig. 3a and 3d. All of the individual antenna elements were connected with coaxial cables to the interface box and then on to the pTx system connectors of the MR scanner.

The phantom used to compare coil performance was an acrylic cylindrical container 180 mm in diameter and 305 mm in height filled with a sucrose doped saline solution [41]. Electromagnetic properties of this solution were measured using a DAKS-12 (SPEAG, Zurich, Switzerland) to be *ε*_r_ = 49 and *σ* = 0.6 S/m. The diameter of the phantom was selected based on the availability of pre-fabricated acrylic tubes that were close in size to that of the human head. The length of phantom was chosen to emulate the human head and neck. The phantom was positioned within the coil formers in a realistic location for the human brain imaging applications.

**Fig. 4.**
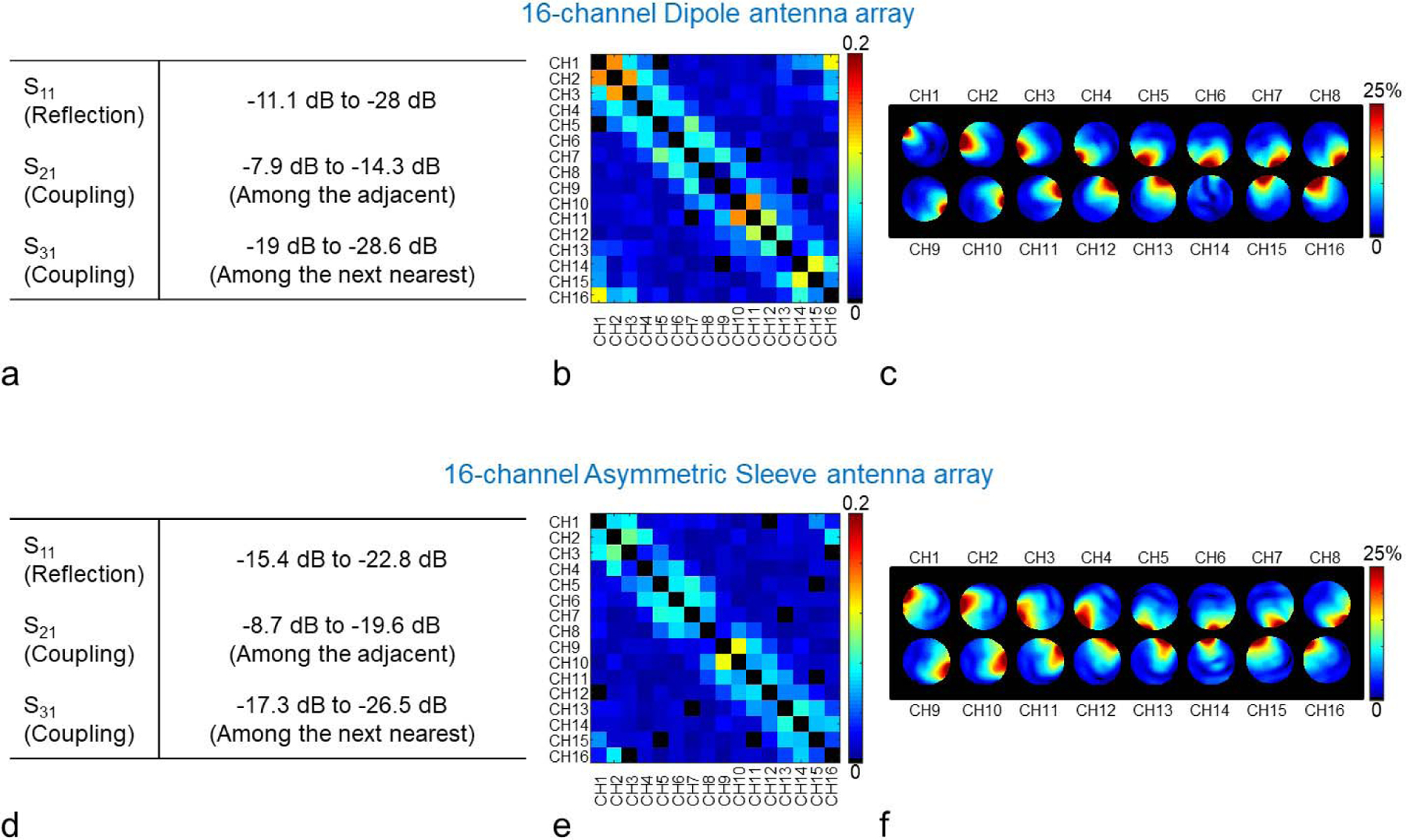
Summary of the achieved S-parameters (a and d), noise covariance matrices (b and e) and individual relative B_1_ magnitude maps (c and f) of the 16-channel dipole (upper row) and the 16-channel asymmetric sleeve antenna (lower row) arrays. Fig. 4c and 4f show relative percentage contribution of each transmitter on each pixel. Note that neither of these radiative arrays have any additional decoupling circuitry. As marked in Fig. 2a and 2c, the transmit field of dipole channel 14 (Fig. 4c), which is positioned over the forehead, appears weaker due to a shifted location and the larger inductors required for this shortened element.

All input reflections and coupling coefficients were measured in bench measurements using a 16-channel network analyzer (ZNBT8, Rohde & Schwarz, Munich, Germany). The S-parameters of all 16 elements both arrays were measured in dB scaled values. The S_11_ of all channels, the S_21_ values with the nearest neighbors, and S_31_ values with the next nearest neighbors of all 16-channel are summarized in Fig. 4a and 4d.

**Fig. 5.**
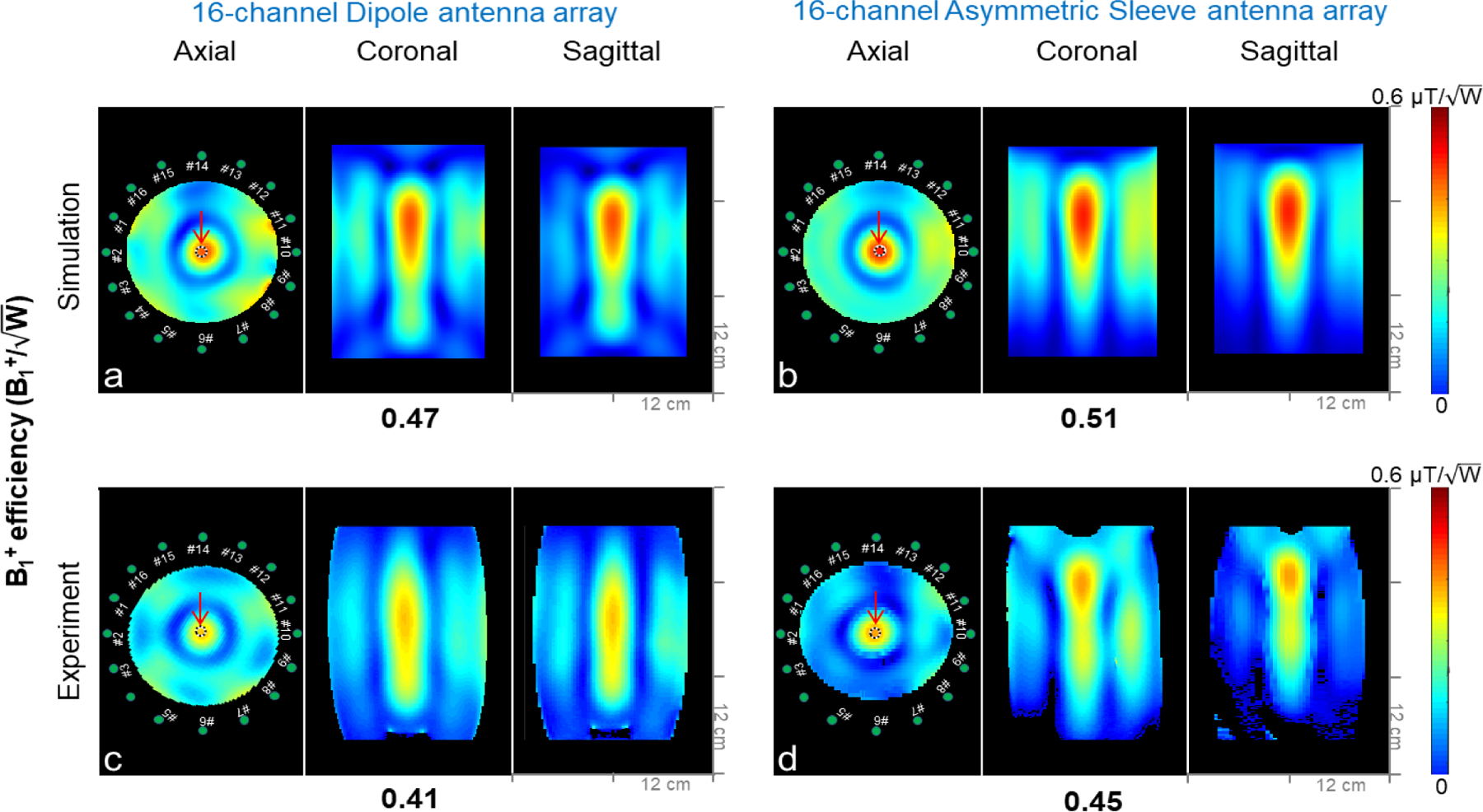
Simulation (a and b) and experimental (c and d) ${\text{B}}_{1}^{+}$ efficiency $\left({\text{B}}_{1}^{+}/\surd \text{W}\right)$ maps of the 16-channel dipole and the 16-channel asymmetric sleeve antenna arrays with a phantom. The results are shown in the axial, coronal, and sagittal planes. For the experimental data (c and d), a threshold was applied for better data display purpose. Note: Due to the reduced interaction among the coaxial cables and antennas of the 16-channel asymmetric sleeve antenna array, the sagittal image shows more field distortion compared to the 16-channel dipole antenna array. Red arrows indicate ROIs where values are measured. The measurements are listed below the corresponding set of figures for comparison. Green dots indicate the position of individual element of the arrays.

Noise covariance matrices of the 16-channel dipole antenna array (Fig. 4b) and the 16-channel asymmetric sleeve antenna array (Fig. 4e) were acquired to experimentally evaluate the crosstalk between the elements [42]. An actual flip angle imaging (AFI) sequence (TR1/TR2 = 20/120 ms, TE = 3.39 ms, nominal flip angle = 60°, GRAPPA (R = 2), resolution = 2 mm × 4 mm × 6 mm) was used to obtain the transmit ${\text{B}}_{1}^{+}$ field maps (Fig. 5c and 5d) with the cylindrical phantom in a circular polarization (CP) mode. All ${\text{B}}_{1}^{+}$ efficiency data sets using AFI were achieved with rectangle pulses (non-selective option and 3D). The flip angle with short TR_1_ and TR_2_ was calculated by

(3) $$\alpha =\arccos\left(\frac{rn-1}{n-r}\right),$$

where *α* = flip angle, n = TR_2_/TR_1_, and $r\approx \frac{1+ncos\alpha }{n+cos\alpha }$ [43]. The flip angle was converted to ${\text{B}}_{1}^{+}$ with

(4) $$\alpha =2\pi \;\gamma \;B_{1}^{+}\tau ,$$

where *γ* is the gyromagnetic ratio and *τ* is the width in seconds of the RF pulse [44]. The individual relative B_1_ magnitude maps corresponding to each channel of the arrays are shown in Fig. 4c for the dipole antenna array and in Fig. 4f for the asymmetric sleeve antenna array. An individual relative B_1_ magnitude map is defined here as the magnitude of each individual transmitter divided by the total magnitude of all sixteen individual transmitter maps. In other words, the individual relative B_1_ map is proportional to the total of all B_1_ fields. The total magnitude of all sixteen individual transmitter maps was obtained by the calculation with the square root sum of squares. High resolution T_2_ weighted TSE images (TR = 5000 ms, TE = 72 ms, TA = 3:45 min, echo train length = 9, BW = 488 Hz/pixel, FOV = 200 mm × 159 mm, resolution = 0.39 mm × 0.39 mm × 1.0 mm) of a human cadaver were obtained for the evaluation of the 16-channel asymmetric sleeve antenna array.

### D. Numerical Simulation

EM simulations (XFdtd, REMCOM, State College, PA, USA) were performed to acquire E- (Fig. 3b) and B- (Fig. 3c) fields of the 250 mm long dipole antenna and E- (Fig, 3e) and B- (Fig.3f) fields of the asymmetric sleeve antenna (200 mm monopole antenna + 50 mm sleeve). These simulations were obtained with parallel alignment of a sleeve (= cable trap) and a coaxial cable for the dipole antenna and with collinear alignment of a sleeve and a coaxial cable for the asymmetric sleeve antenna. Coaxial cables were modeled by parallel cylindrical central bars and pipe structures with realistic dimensions and electrical characteristic of copper. All cable traps were modeled and included in the simulation to match the experimental setup as much as possible.

**Fig. 6.**
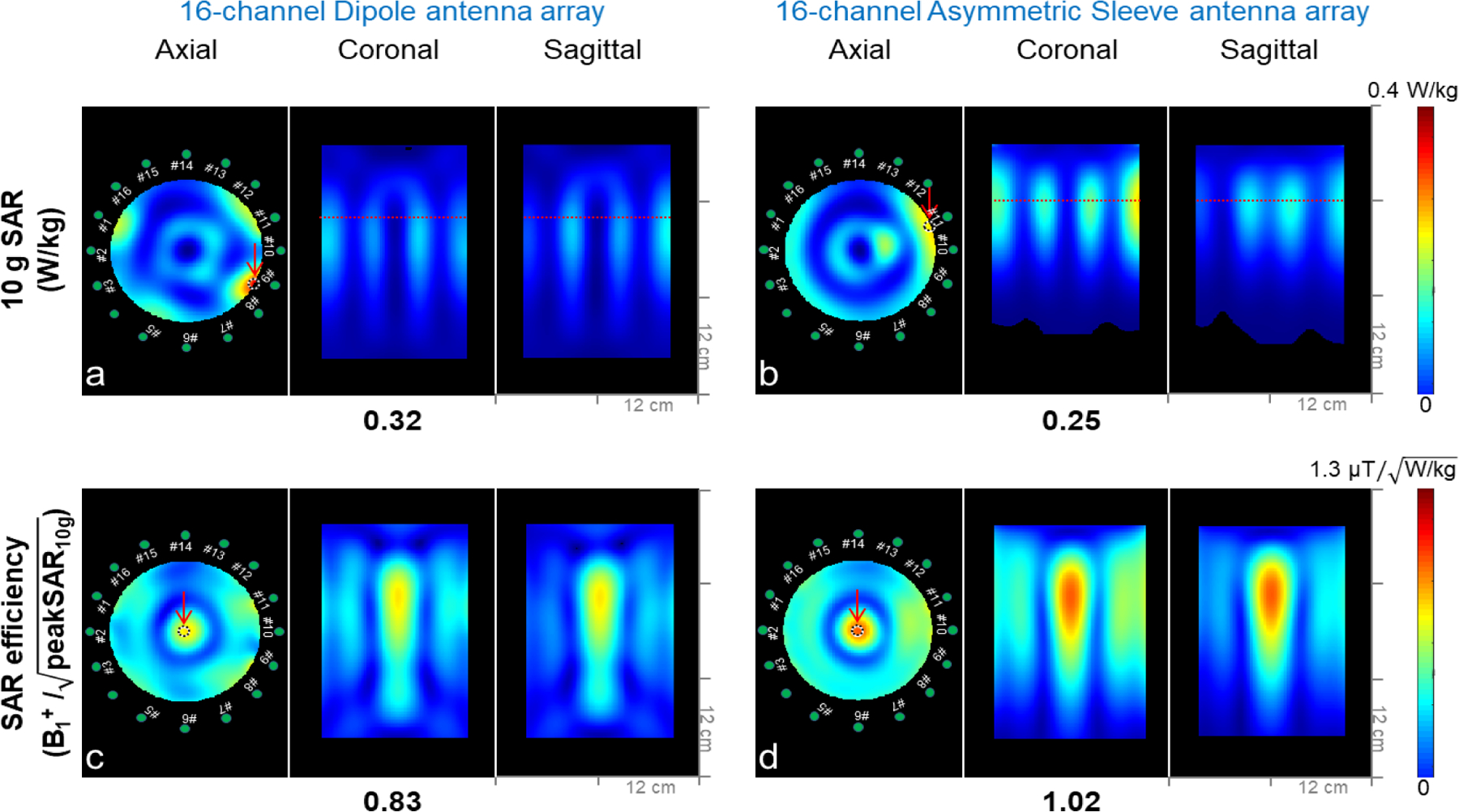
10 g SAR (a and b) and SAR efficiency $\left({\text{B}}_{1}^{+}/{\sqrt{\;}}_{\text{peak}}\text{SA}{\text{R}}_{10\text{g}}\right))$ (c and d) maps of the 16-channel dipole and asymmetric sleeve antenna arrays with a phantom; results are shown in axial, coronal, and sagittal planes. Red dotted lines of a coronal and a sagittal plane in Fig 6a and 6b indicate the location of the axial plane with peak 10 g SAR. Red arrows indicate ROIs where values are measured. The measurements are listed below the corresponding set of figures for comparison.

EM simulations were also used to calculate ${\text{B}}_{1}^{+}$ fields (Fig. 5a and 5b) and 10 g SAR (Fig. 6a and 6b) of both the 16-channel dipole antenna and the asymmetric sleeve antenna arrays with non-isotropic gridding (minimum: 4 mm and maximum: 8 mm). A phantom with matching dimensions and electrical properties was selected for experimental verification with a 1 mm × 1 mm × 1 mm resolution (re-gridded by post-processing). Importantly, all of the simulated 16-channel arrays included the coaxial cables and the sleeve - thus closely resembling the practical coil setup. All data were calculated using MATLAB (The Mathworks, Inc., Natick, MA, USA) after EM simulation. ${\text{B}}_{1}^{+}$ fields were determined from

(5) $$B_{1}^{+}=\left|\frac{B_{x}+i\;B_{y}}{2}\right|,$$

where B_x_ and B_y_ are the complex amplitudes of x- and y-oriented RF magnetic fields, respectively [45].

${\text{B}}_{1}^{+}$ efficiency, 10 g SAR, and SAR efficiency maps shown below (Fig. 5 and Fig. 6) compare the two 16-channel arrays. ${\text{B}}_{1}^{+}$ fields were normalized to 1 W, in order to evaluate the ${\text{B}}_{1}^{+}$ efficiency. The normalization was performed over the power supplied to the all antenna elements. For the safety validation, 10 g SAR (W/kg) values were calculated from the E-field and compared between arrays. SAR efficiency values, which are ${\text{B}}_{1}^{+}$ efficiency per square root of peak 10 g SAR, were compared between arrays as shown in Fig. 6c and 6d. For a quantitative comparison, the highest ${\text{B}}_{1}^{+}$ efficiency, 10 g SAR, and SAR efficiency areas are indicated in the axial plane of the arrays. The values of each ROI (2 mm isotropic voxel) are indicated below the figures.

## III. Results

### A. Comparison of Bench Measurements, Simulation, and Experiments Between the 16-Channel Arrays

Bench measurements of the scattering parameters for the reflection (S_11_) and coupling (S_21_, and S_31_) coefficients when the coils were loaded with a uniform cylindrical phantom are summarized in Fig. 4a and 4d for both arrays, respectively. The S_11_ values of the 16-channel dipole and the asymmetric sleeve antenna arrays ranged between −11.1 dB to −28 dB, and between −15.4 dB to −22.8 dB, respectively. Coupling between adjacent elements (S_21_) was in the range of −7.9 dB to −14.3 dB for the dipole antenna array and −8.7 dB to −19.6 dB for the asymmetric sleeve antenna array. Noise covariance matrices were obtained in an MR experiment and are shown in Fig. 4b and 4e. A maximum correlation value of 0.17 was observed for the 16-channel dipole antenna array and of 0.11 for the 16-channel asymmetric sleeve antenna array, respectively. Overall ${\text{B}}_{1}^{+}$ efficiency loss due to the inter-element coupling was calculated and shown to be 44 % for the 16-channel dipole antenna array and 22.7 % for the 16-channel asymmetric sleeve antenna array, respectively.

The ${\text{B}}_{1}^{+}$ efficiency comparisons between the two arrays calculated from electromagnetic simulations and obtained experimentally are illustrated in Fig. 5. Compared to the 16-channel dipole antenna array, the 16-channel asymmetric sleeve antenna array achieved ∼8 % higher ${\text{B}}_{1}^{+}$ (center) efficiency both in simulation and experimentally, as measured in the indicated region of interest (ROI) in the phantom. Red arrows indicate ROIs.

The overall average and peak values of SAR and 10 g SAR were summarized in Table I. Peak 10 g SAR values of the 16-channel dipole (Fig. 6a) and the 16-channel asymmetric sleeve antenna arrays (Fig. 6b) were 0.32 W/kg and 0.25 W/kg with the phantom, respectively. This indicates 28 % lower SAR value for the 16-channel asymmetric sleeve antenna array compared to the 16-channel dipole antenna array. As observed in the axial plane, the generated B- and E- fields among coaxial cables and antenna elements of the 16-channel dipole antenna array led to higher peak 10 g SAR in the periphery area of the phantom compared to the 16-channel sleeve antenna array. The result is that the 16-channel asymmetric sleeve antenna array showed 18.6 % higher SAR efficiency compared to the 16-channel dipole antenna array in the simulation due to the lower peak 10 g SAR values depicted in Fig. 6c and 6d.

**Fig. 7.**
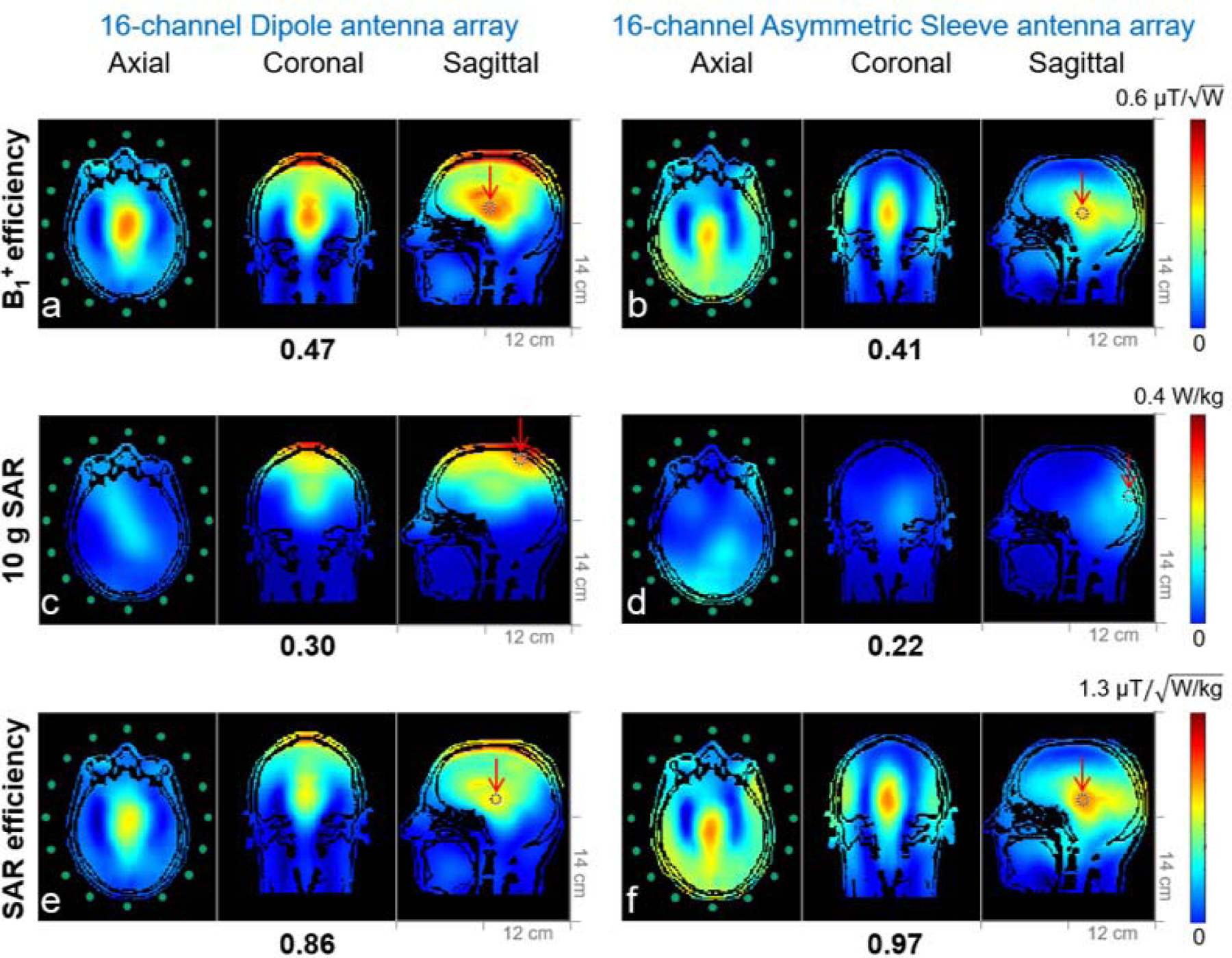
Simulation of ${\text{B}}_{1}^{+}$ efficiency (a and b), 10 g SAR (c and d) and SAR efficiency (e and f) maps of the 16-channel dipole and the 16-channel asymmetric sleeve antenna arrays with a human head model (Duke) in an axial, coronal, and sagittal planes. The peak 10 g SAR of the 16-channel asymmetric sleeve antenna array (d) is substantially lower compared to the 16-channel dipole antenna array (c). SAR efficiency of the 16-channel asymmetric sleeve antenna array (f) is higher compared to the 16-channel dipole antenna array (e).

Fig. 7 shows a ${\text{B}}_{1}^{+}$ efficiency, 10 g SAR and, SAR efficiency comparison of the 16-channel arrays in electromagnetic simulations with a human model (Duke). As indicated in Fig. 7a and 7b, the ${\text{B}}_{1}^{+}$ efficiency of the 16-channel dipole antenna array was generally higher compared to values achievable with the 16-channel asymmetric sleeve antenna array. For both arrays the human head model loaded the antennas heavier in the anterior-posterior axis compared to the phantom and this heavy loading helped to reduce the coupling between antennas and coaxial feed cables with the dipole antenna array.

To avoid alignment of the central dipole feed point with the eyes, the human head model was shifted down from the central feed point towards the lower leg side of the dipole antenna and this results in a shift of the peak 10 g SAR more to the superior part of the brain. Peak 10 g SAR values of the 16-channel dipole (Fig. 7c) and the 16-channel asymmetric sleeve antenna (Fig. 7d) arrays are 0.30 W/kg and 0.22 W/kg with the human head model, respectively. This equates to 36.4 % lower peak SAR for the 16-channel asymmetric sleeve antenna array compared to the 16-channel dipole antenna array.

### B. Human Cadaver Experiments With the 16-Channel Asymmetric Sleeve Antenna Array

**Fig. 8.**
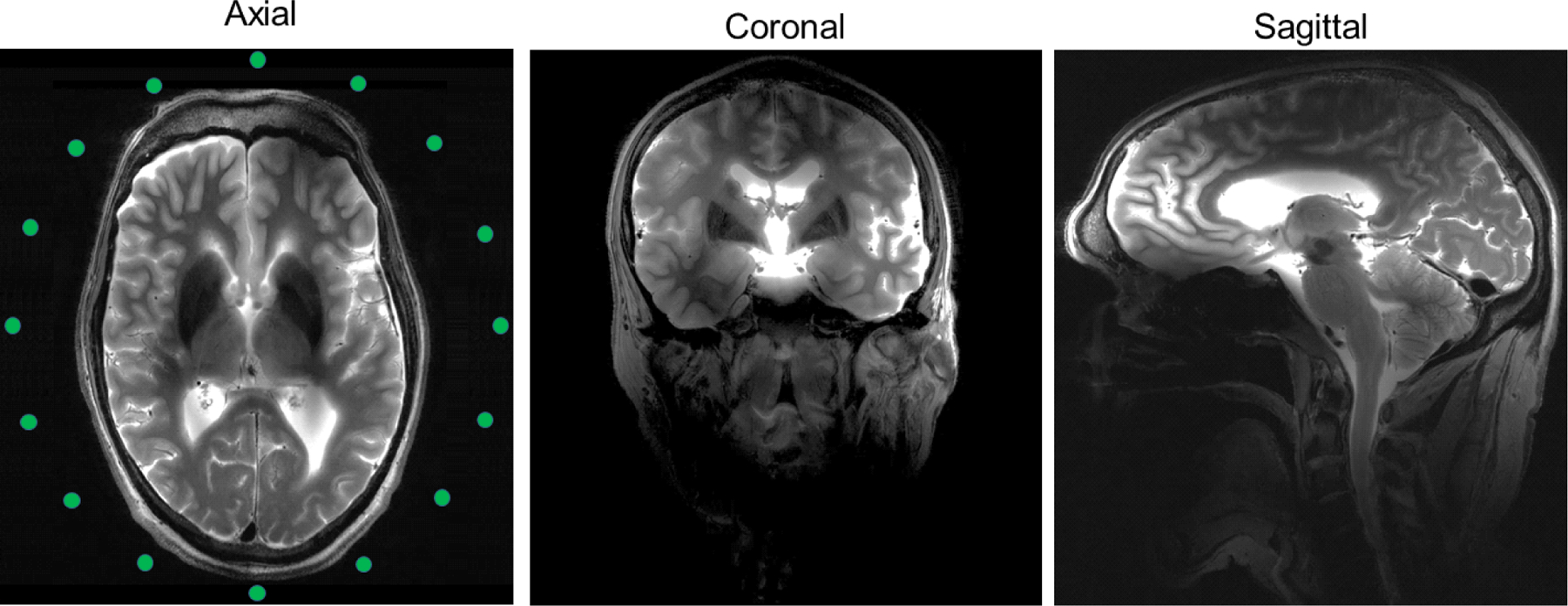
Turbo-spin-echo (TSE) images acquired at 10.5 T with the 16-channel asymmetric sleeve antenna array with human cadaver in the axial, coronal and sagittal planes. The displayed images were achieved in circular polarization (CP) mode without any B_1_ shimming or pTx pulse optimization technique. TR = 5000 ms, TE = 72 ms, TA = 3:45 min, BW = 488 Hz/pixel, FOV = 200 mm × 159 mm, resolution = 0.39 mm × 0.39 mm × 1.0 mm.

The high resolution turbo-spin-echo (TSE) human cadaver images acquired at 10.5 T, shown in Fig. 8, demonstrate the good overall B_1_ penetration and field distribution of the 16-channel asymmetric sleeve antenna array. These images were achieved with the 16-channel asymmetric sleeve antenna array driven in a CP mode without any further B_1_ shimming or pTx pulses adjustments for improved transmit B_1_ uniformity; the images were also not manipulated with reconstruction techniques aimed at flattening the signal intensity variations. In axial images, the signal intensity is highly uniform even in the absence of specific efforts to improve its uniformity.

## IV. Discussion

Within the described parameters of our comparison, our results demonstrate clear benefits of the asymmetric sleeve antenna concept compared to dipole antenna array when used as RF coil array element at 447 MHz. The structure of the sleeve antenna itself closely resembles the dipole antenna with one important difference: the layout of a sleeve antenna element and the coaxial feed cable are collinear, whereas in the dipole antenna, the feed cable attaches at the center of the antenna and necessarily runs parallel to a portion of the dipole antenna to exit the coil assembly. The dipole antenna thus suffers from E- and B-fields interactions between the antenna element and the parallel-running feed cable. In MR body applications, dipole antennas can be placed directly on the body resulting in high coupling to the sample. This reduces interaction between the dipole antennas and coaxial feed cables. Remaining unbalanced currents flowing on coaxial feed cables can be tackled using balun matching networks, cable traps or the combination of the two [34], [35], [46]. For head applications, however, the weaker coupling combined with coaxial cable routing does affect both the E- and B-fields pattern of a dipole antenna. The collinear arrangements of the elements that make up the asymmetric sleeve antenna minimize these E- and B-fields interactions.

Moreover, the floating cable trap design of a sleeve antenna acts to further suppress imbalanced RF currents leaking onto the outer surface of the shield of the coaxial feed cable. These floating cable traps did not negatively influence either the antenna efficiency nor the radiation pattern.

The other important advantage of the asymmetric sleeve antenna array, compared to the dipole antenna array, is substantially lower peak 10 g SAR, and resulting enhanced SAR efficiency over a central ROI. To evaluate the safety at UHF, 10 g SAR can be used as an important criterion to estimate local E-fields and the related temperature changes [8], [47]–[49]. As shown in Fig. 7,^1^ the peak 10 g SAR of the 16-channel asymmetric sleeve antenna array loaded with the human model was substantially lower than that of the 16-channel dipole antenna array. As a result, asymmetric sleeve antenna concepts allow for improved RF transmission for 10.5 T human brain imaging (for a given number of transmits channels).

Recently a passive feeding network and a snake type antenna structure were suggested to reduce peak 10 g SAR by Zivkovic *et al.* [50] and Steensma *et al.* [51], respectively. In future work, we will evaluate and compare SAR and SAR efficiency as a transceiver with these newly suggested techniques and we will also extend the comparison towards transmit-only and receive-only (TORO) arrays [52].

As indicated in Fig. 5d, experimentally for CP excitation, the asymmetric sleeve antenna array showed less uniform ${\text{B}}_{1}^{+}$ efficiency distribution in the axial slice compared to the dipole antenna array. In future work, we will evaluate the achievable uniformity beyond simple CP excitation at 10.5 T while utilizing parallel transmission within safe imaging parameters.

We also observed non-uniformity along the superior-inferior (z-) direction, visible in the sagittal image (Fig. 8) produced by the asymmetric sleeve antenna array. This is to be expected as the cylindrical layout of the antenna elements does not allow for antenna conductors to be in the vicinity of the superior portion of the head. However, this could possibly be remedied using monopole elements that are not straight, but conform to the curvature of the head in the z-direction, and we will evaluate this in future work. Furthermore, B_1_ shimming using pTx techniques can be utilized to improve the uniformity of images encompassing the entire human head, and we plan to evaluate this in future work.

## V. Conclusion

Here we present an asymmetric sleeve antenna concept for UHF MRI of the human head and demonstrate the potential of this antenna type for imaging at 10.5 T. For future in-vivo human brain imaging, the asymmetric sleeve antenna array will be further optimized regarding number of channels and more form fitting geometry. This optimized sleeve antenna array will be RF safety validated for in vivo human head experiments and carefully evaluated for whole head shim capability. It is expected that the optimized sleeve antenna array geometry and utilization of pTx techniques will support further improved B_1_ fields and allow for lower peak 10 g SAR, which could lead to new neurological developments using UHF MRI.

## Supplementary Material

supp1-3047354

## Figures and Tables

**TABLE I:** Average and Peak Values of SAR and 10 g SAR of the 16-Channel Dipole and Asymmetric Sleeve Antenna Arrays

	16-channel Dipole	16-channel Asymmetric Sleeve antenna

Average SAR (W/kg)	0.067	0.052
Peak SAR (W/kg)	0.45	0.31
Average 10 g SAR (W/kg)	0.091	0.085
Peak 10 g SAR (W/kg)	0.32	0.25

## Footnotes

1 A supplementary figure is available in the supporting documents.
