# Supplementary figures and images for: Comparison of 16-Channel Asymmetric Sleeve Antenna and Dipole Antenna Transceiver Arrays at 10.5 Tesla MRI
Source: IEEE Trans Med Imaging. Author manuscript; Available in PMC 2021 Apr 27. (PMC8078892; doi:10.1109/TMI.2020.3047354)

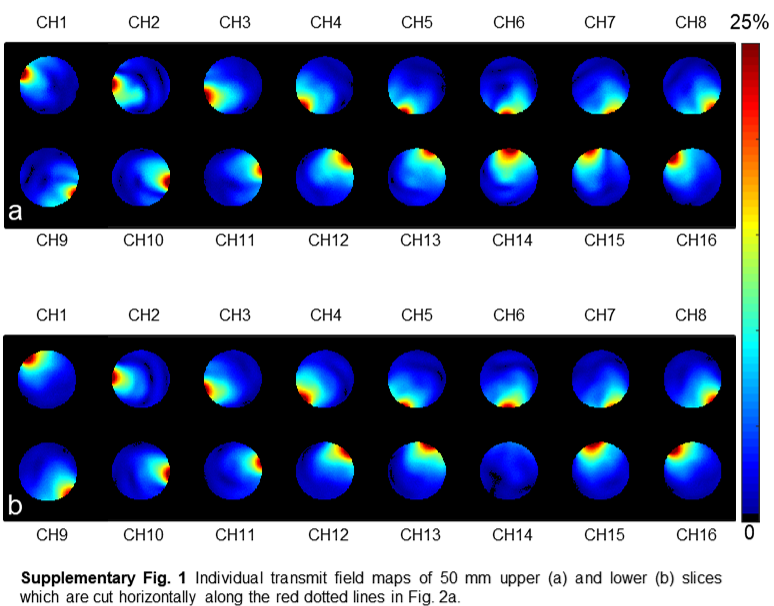

Supplement: supp1-3047354 [file NIHMS1689912-supplement-supp1-3047354.png]
